# Supplementary material for: Genome-Wide Association Mapping for Yield and Yield-Related Traits in Rice (Oryza Sativa L.) Using SNPs Markers
Source: Genes (Basel). 2023 May 15;14(5):1089. doi: 10.3390/genes14051089 (PMC10218429; doi:10.3390/genes14051089)
Supplement: Supplementary file 1 [file genes-14-01089-s001.zip › genes-2324582-supplementary.pdf]

<u>Supplementary File</u>

Supplementary Table S1

S.No	Variety Name	Origin	Ecotype	Group	S.No	Variety Name	Origin	Ecotype	Group
1	Lmont	USDA, USA	*Indica*	Landrace	51	ROXERO REGUE	USDA, USA	*Indica*	Landrace
2	Bond	USDA, USA	*Indica*	Landrace	52	H-256-76-1-1-1	USDA, USA	*Indica*	Landrace
3	IR-36	USDA, USA	*Indica*	Landrace	53	PALMAN	USDA, USA	*Indica*	Landrace
4	Delvex	USDA, USA	*Indica*	Landrace	54	JASMINE-85	Thailand	*Indica*	Landrace
5	Teqing	USDA, USA	*Indica*	Landrace	55	A-301	USDA, USA	*Indica*	Landrace
6	Nira	USDA, USA	*Indica*	Landrace	56	L-202	USDA,USA	*Indica*	Landrace
7	Cica	USDA, USA	*Indica*	Landrace	57	VE GOLD	USDA, USA	*Indica*	Landrace
8	Koshihikari	USDA, USA	*Indica*	Landrace	58	L-203	USDA, USA	*Indica*	Landrace
9	IR-64	USDA, USA	*Indica*	Landrace	59	IR-6	Pakistan	*Indica*	Land Race
10	Bellmont	USDA, USA	*Indica*	Landrace	60	SATHI BASMATI	Pakistan	*Indica*	Landrace
11	Yangzi-95	USDA, USA	*Indica*	Landrace	61	SHAHEEN BASMATI	Pakistan	*Indica*	Landrace
12	Gui-99	USDA, USA	*Indica*	Landrace	62	BASMATI-198	Pakistan	*Indica*	Landrace
13	L-203	USDA, USA	*Indica*	Landrace	63	BASMATI-370	Pakistan	*Indica*	Landrace
14	CE-65	USDA, USA	*Indica*	Landrace	64	BASMATI-Pak	Pakistan	*Indica*	Landrace
15	Zao-40	USDA, USA	*Indica*	Landrace	65	BASMATI-385	Pakistan	*Indica*	Landrace
16	LA-110	USDA, USA	*Indica*	Landrace	66	BASMATI-515	Pakistan	*Indica*	Landrace
17	Rando	USDA, USA	*Indica*	Landrace	67	R-456	Pakistan	*Indica*	IV
18	Jakson	USDA, USA	*Indica*	Landrace	68	CB-5	Pakistan	*Indica*	ABL
19	WC-4644	USDA, USA	*Indica*	Landrace	69	CB-10	Pakistan	*Indica*	ABL
20	Tsai Yuan Chung	USDA, USA	*Indica*	Landrace	70	CB-11	Pakistan	*Indica*	ABL
21	Cica-6	USDA, USA	*Indica*	Landrace	71	CB-12	Pakistan	*Indica*	ABL
22	IR-456-3-2-1	USDA, USA	*Indica*	Landrace	72	CB-13	Pakistan	*Indica*	ABL
23	Newbonnet	USDA, USA	*Indica*	Landrace	73	CB-14	Pakistan	*Indica*	ABL
24	Newrex	USDA, USA	*Indica*	Landrace	74	CB-15	Pakistan	*Indica*	ABL
25	Taichung Native-1	USDA, USA	*Indica*	Landrace	75	CB-16	Pakistan	*Indica*	ABL
26	SinumPaga Selection	USDA, USA	*Indica*	Landrace	76	CB-17	Pakistan	*Indica*	ABL

27	Taducan	USDA, USA	*Indica*	Landrace	77	CB-19	Pakistan	*Indica*	ABL
28	Dee Geo Woo Gen	USDA, USA	*Indica*	Landrace	78	CB-20	Pakistan	*Indica*	ABL
29	Stg-663228	USDA, USA	*Indica*	Landrace	79	CB-209	Pakistan	*Indica*	ABL
30	Lebonnet	USDA, USA	*Indica*	Landrace	80	CB-21	Pakistan	*Indica*	ABL
31	Starbonnet	USDA, USA	*Indica*	Landrace	81	CB-22	Pakistan	*Indica*	ABL
32	Della	USDA, USA	*Indica*	Landrace	82	CB-26	Pakistan	*Indica*	ABL
33	Toro-2	USDA, USA	*Indica*	Landrace	83	CB-27	Pakistan	*Indica*	ABL
34	Delitus	USDA, USA	*Indica*	Landrace	84	CB-28	Pakistan	*Indica*	ABL
35	Dellrose	USDA, USA	*Indica*	Landrace	85	CB-29	Pakistan	*Indica*	ABL
36	CDR-448	USDA, USA	*Indica*	Landrace	86	CB-30	Pakistan	*Indica*	ABL
37	CDR-201	USDA, USA	*Indica*	Landrace	87	CB-31	Pakistan	*Indica*	ABL
38	B5-Xiequizao	USDA, USA	*Indica*	Landrace	88	CB-32	Pakistan	*Indica*	ABL
39	L-203	USDA, USA	*Indica*	Landrace	89	CB-33	Pakistan	*Indica*	ABL
40	VeGold	USDA, USA	*Indica*	Landrace	90	CB-34	Pakistan	*Indica*	ABL
41	TP-49	USDA, USA	*Indica*	Landrace	91	CB-36	Pakistan	*Indica*	ABL
42	Hill Long Grain	USDA, USA	*Indica*	Landrace	92	CB-38	Pakistan	*Indica*	ABL
43	L-202	USDA, USA	*Indica*	Landrace	93	CB-39	Pakistan	*Indica*	ABL
44	A-301	USDA, USA	*Indica*	Landrace	94	CB-40	Pakistan	*Indica*	ABL
45	L-202	USDA, USA	*Indica*	Landrace	95	CB-41	Pakistan	*Indica*	ABL
46	V-203	USDA, USA	*Indica*	Landrace	96	CB-43	Pakistan	*Indica*	ABL
47	PALMAN	USDA, USA	*Indica*	Landrace	97	CB-44	Pakistan	*Indica*	ABL
48	87-1-550	USDA, USA	*Indica*	Landrace	98	KSK-282	Pakistan	*Indica*	ABL
49	79	USDA, USA	*Indica*	Landrace	99	KSK-133	Pakistan	*Indica*	ABL
50	923	USDA, USA	*Indica*	Landrace	100	ROXERO REGUE	USDA, USA	*Indica*	Landrace

Supplementary Table S2. Phenotypic trait measurement procedure.

S. no	Phenotypic trait	Procedure for measurement
1	Plant height (cm) (PH)	Plant height was measured for five plants per variety per replication. The height of these five selected plants measured from the end of the shoot to the tip of the central stem when the plant was fully matured. The average height was calculated and expressed in centimeters (cm).
2	Number of tillers per plant (TP)	The total number of tillers per plant in five randomly selected plants from each variety and each replication was taken at harvest. Their mean was estimated and expressed in the number of tillers per plant.
3	Days to maturity (DM)	The sum of days taken from transplanting to the harvesting of the plants in a plot was recorded for each variety as days to maturity.
4	Days to 50 percent flowering (DF)	A sum of days, from transplanting to the flowering of 50 percent of the plants in a plot was recorded for each variety as days to 50 percent flowering (DF).
5	Panicle length (cm) (PL)	Three mature panicles from the selected plants were taken. Panicle length was estimated as the distance from the panicle neck to the tip of the panicle in centimeters (cm).
6	Number of grains per panicle (G/P)	The number of grains in panicles of selected plant (3 plants) were calculated and documented.
7	Number of unfilled grains per panicle (UG/P)	Number of unfilled grain from selected panicle from all three plants were counted and mentioned as unfilled grain per panicle
8	Seed setting %age (SS)	Total filled and empty grains have been calculated. Seed setting %agewas estimated as $\text{Seed Setting \%age} = \frac{\text{Number of filled grains}}{(\text{Number of filled grains} + \text{number of unfilled grains})} x100$
9	Thousand grain weight (g) (TGW)	Seeds were taken from the sample and weighed as 1000 seed weight
10	Yield per plot (kg/plot) (Y/P)	0.5 m^2 was harvested for measurement of yield components. Harvested plants were threshed and sun-dried. Total grain weight for each plot was measured. All the grains from the replication were collected, sun-dried (at 14% moisture) and cleaned. The weight of the grains of these plants was calculated with an electric balance. Plants were collected from the middle of all rows, leaving 0.5 m of the row at the ends. Therefore, the total harvest area was 1.5 m^2.
11	Yield per hectare (kg/ha) (Y/H)	Yield per plot was calculated in kilograms per hectare (kg/ha) at 14% moisture content. After measuring the yield component, the grain yield from that 0.5 m^2 area was added with the total grain yield. Grain yield Kg/ha was calculated by the formula $\text{Grain yield (kg/ha)} = \frac{(\text{GYP}/0.8)\ \text{X}\,1000}{10000}$

Supplementary Table S3. Mean squares values of studied traits during season 1.

S.O.V	D.F	DF	DM	PH	TP	PL	G/P	UG/P	SS	TGW	Y/P	Y/H
Replications	2	1.97	4.61	417.46	5.77	4.17	150.28	343.26	108.39	1.26	2.33	243012
Genotypes	99	9.93*	7.03*	566.81**	34.19**	11.46**	1917.32**	417.93**	102.43**	40.34**	1.07**	3591471**
Error	198	6.28	3.88	31.06	4.22	2.24	648.94	120.83	23.43	5.3	0.41	156805

Level of significance p<0.05=* and p<0.01=** *= Significant, ** Highly Significant

DF= Days to 50% Flowering, DM = Days to Maturity, PH= Plant Height (cm), TP= Tiller per Plant, PL= Panicle Length (cm), G/P= Grains per Panicle, UG/P= Unfilled grains/Panicle, SS= Seed Setting percentage, TGW= 1000 Grains weight (g), Y/P=Yield/plot (kg), Y/H= Yield/hectare (Kg)

Supplementary Table S4. Principal components (PCs) for 11 agronomic traits of hundred genotypes of rice.

	PC 1	PC 2	PC 3	PC 4	PC 5	PC 6	PC 7	PC 8	PC 9	PC 10
**TP**	0.044	0.110	-0.038	0.405	**0.879**	0.147	0.096	-0.053	-0.121	-0.014
**DF**	-0.007	0.012	0.015	-0.052	-0.190	**0.818**	0.320	0.200	-0.385	-0.029
**PH**	0.144	**0.780**	**0.604**	-0.035	-0.057	-0.029	-0.018	-0.005	-0.002	0.001
**PL**	0.015	-0.021	0.024	-0.265	0.103	-0.434	**0.809**	0.232	-0.146	0.015
**GP**	**0.984**	-0.116	-0.091	-0.042	-0.028	0.000	-0.020	-0.062	-0.054	-0.011
**UG/P**	0.043	-0.545	0.715	0.138	0.076	0.073	0.001	0.298	0.265	0.015
**SS**	0.075	0.254	-0.327	-0.015	0.060	0.127	-0.025	**0.689**	**0.569**	0.064
**TGW**	0.032	0.062	-0.075	**0.856**	-0.409	-0.142	0.256	0.023	0.011	-0.056
**Yield kg/Plot**	0.007	-0.003	0.004	0.052	-0.023	0.026	0.031	-0.070	-0.025	0.995
**MD**	0.005	0.015	-0.002	-0.075	-0.005	0.277	0.408	-0.575	0.647	-0.040

Supplementary Table S5. Mean squares values of studied traits during season 2.

**S.O.V**	**D.F**	**DF**	**DM**	**PH**	**TP**	**PL**	**G/P**	**UG/P**	**SS**	**TGW**	**Y/P**	**Y/H**
Replications	2	92.92	102.79	55.07	34.74	39.60	256.86	14.32	67.46	123.91	2.44	432927
Genotypes	99	11.00**	5.34*	342.00**	35.58**	9.55**	1596.58**	260.53**	100.18**	39.73**	0.90**	3519407**
Error	198	4.49	3.81	85.54	3.07	1.95	696.77	124.43	20.01	4.18	0.41	123622

Level of significance p<0.05=* and p<0.01=** *= Significant, ** Highly Significant

DF= Days to 50% Flowering, DM = Days to Maturity, PH= Plant Height (cm), TP= Tiller per Plant, PL= Panicle Length (cm), G/P= Grains per Panicle, UG/P= Unfilled grains/Panicle, SS= Seed Setting percentage, TGW= 1000 Grains weight (g), Y/P=Yield/plot (kg), Y/H= Yield/hectare (Kg)

Supplementary Table S6

**Traits**	SNP	Chromosome	Position	P.value	R%
**Days to 50% flowering (DF)**	OsGRb30080	2	18212087	0.000103702	19.84
	OsGRb14914	7	20814664	0.000112756	19.66
	OsGRb15039	7	22304042	0.000112756	19.66
	OsGRb05211	2	29829169	0.000115539	19.61
	OsGRb24510	2	9826527	0.000127307	19.39
	OsGRb24572	2	12405692	0.000148829	19.05
	OsGRb24900	2	30132327	0.000192517	18.49
	OsGRb04151	2	17990840	0.000207459	18.33
	OsGRb04250	2	19120460	0.000240121	18.01
	OsGRg02806	2	17273659	0.000248846	17.93
	OsGRb04064	2	16775829	0.000254602	17.88

	OsGRb04108	2	17345198	0.000282383	17.66
	OsGRb04456	2	21810979	0.000314737	17.43
	OsGRb04559	2	22869364	0.000314737	17.43
	OsGRb04569	2	23120515	0.000314737	17.43
	OsGRb30142	2	24285137	0.00034923	17.20
	OsGRb04511	2	22354743	0.000358582	17.15
	OsGRb16114	8	8615206	0.000426811	16.78
	OsGRb16145	8	8929911	0.000449302	16.67
	OsGRb04104	2	17309380	0.000512281	16.39
	OsGRb04421	2	20975538	0.000519788	16.36
	OsGRb04893	2	26259806	0.000555053	16.22
	OsGRb21146	11	15034296	0.000563352	16.19
	OsGRg02884	2	19568531	0.000566321	16.18
	OsGRg03095	2	23712290	0.000566321	16.18
	OsGRb25451	3	24409509	0.000582683	16.12
	OsGRb21461	11	19084575	0.000582683	16.12
	OsGRg14009	11	18935157	0.000582683	16.12
	OsGRb05101	2	28940961	0.000596317	16.07
	OsGRg02717	2	11885546	0.000596317	16.07
	OsGRb04072	2	16904305	0.000600264	16.06
	OsGRb04943	2	26640578	0.000631732	15.95
	OsGRg03565	2	30862290	0.000725216	15.66
	OsGRb21548	11	19994753	0.000735707	15.63
	OsGRg02708	2	11594325	0.000752687	15.58
	OsGRb25462	3	25181277	0.000767637	15.54
	OsGRb25459	3	25131417	0.000772959	15.53
	OsGRg05363	3	24961621	0.000772959	15.53
	OsGRb21468	11	19129697	0.000772959	15.53
	OsGRb21471	11	19170789	0.000772959	15.53
	OsGRb32442	11	19026652	0.000772959	15.53
	OsGRg03058	2	23529294	0.000791106	15.48
	OsGRg03325	2	26931234	0.000831996	15.37
	OsGRb30127	2	22416322	0.000839333	15.35
	OsGRb04643	2	23850363	0.000880233	15.25
	OsGRg02730	2	12762219	0.000883595	15.25
	OsGRb24567	2	12345631	0.000900866	15.21

	OsGRg13980	11	18173691	0.000941609	15.11
	OsGRb25578	3	33753141	0.000971774	15.05
**Maturity days (DM)**	OsGRb09564	4	27939281	0.000827828	12.97
	OsGRg04275	3	4267961	0.000110491	12.35
	OsGRb17703	9	11125119	0.000159335	11.57
**No. of grain/panicle (G/P)**	OsGRb28603	9	12952275	0.000252418	20.79
	OsGRb31905	9	17074373	0.000256058	20.76
	OsGRb17510	9	7800022	0.00035416	20.08
	OsGRb28600	9	12915373	0.000413351	19.76
	OsGRg12308	9	14048196	0.000532384	19.24
	OsGRg12310	9	14048809	0.00055683	19.14
	OsGRb18178	9	19105152	0.000694254	18.69
	OsGRb27733	7	20438541	0.000870925	18.23
**Plant height (PH)**	OsGRb14446	7	14594194	2.02E-06	25.55
	OsGRb30717	4	29956594	5.59E-05	17.27
	OsGRb09705	4	29950172	0.000117035	15.52
	OsGRb31240	7	9363018	0.000119296	15.47
	OsGRb12976	6	16465767	0.000121861	15.42
	OsGRb13852	7	485365	0.000134253	15.20
	OsGRb13891	7	754356	0.000134253	15.20
	OsGRg01923	1	39950738	0.000256562	13.70
	OsGRb13812	7	219361	0.000418686	12.58
	OsGRb14044	7	1929142	0.000492237	12.21
	OsGRb28184	8	14792948	0.000524592	12.07
	OsGRb27102	6	24322609	0.0005983	11.77
	OsGRb27139	6	25535681	0.0005983	11.77
	OsGRg05238	3	21673888	0.000648167	11.60
	OsGRb14503	7	15365358	0.000664937	11.54
	OsGRb13785	7	20400	0.000686431	11.47
**Panicle Length (PL)**	OsGRb23906	1	10116371	0.000191598	18.77
	OsGRg09425	6	21482221	0.000860313	15.54
	OsGRb24377	1	40794243	0.000190652	13.89
	OsGRb10830	5	10350550	0.000257357	13.27
**Seed Setting %age (SS)**	OsGRb30591	4	12914840	3.70464E-05	22.69
	OsGRb08881	4	13725269	0.000195545	18.97
	OsGRb08538	4	4440931	0.000246253	18.46

	OsGRg06306	4	11320635	0.000258291	18.36
	OsGRb25787	4	12725098	0.00026478	18.31
	OsGRb09155	4	19209543	0.000283155	18.16
	OsGRb08910	4	14035735	0.000287425	18.13
	OsGRb25664	4	8124043	0.000302411	18.02
	OsGRb09180	4	19850601	0.00030797	17.98
	OsGRg06407	4	19601384	0.000626121	17.90
	OsGRg06430	4	19893385	0.000626121	17.88
	OsGRg06261	4	6968387	0.000653867	17.82
	OsGRb25862	4	19570740	0.000655505	17.77
	OsGRb09200	4	20060586	0.000665116	17.77
	OsGRb09238	4	20485782	0.000665116	17.70
	OsGRb09240	4	20505437	0.000665116	17.67
	OsGRg06458	4	20426851	0.00067696	17.66
	OsGRb08754	4	11300762	0.000678377	17.56
	OsGRg06304	4	11316471	0.000678377	17.49
	OsGRb25740	4	10304323	0.000683696	17.45
	OsGRb08742	4	10624962	0.000688745	17.39
	OsGRb25839	4	17182231	0.000690069	17.36
	OsGRg06490	4	21414906	0.000703709	17.36
	OsGRg06518	4	22025935	0.000718576	17.34
	OsGRb17738	9	11674055	0.0007187	17.33
	OsGRg11329	8	3389509	0.00072166	17.32
	OsGRb25742	4	10354477	0.000732621	17.32
	OsGRb09195	4	20025177	0.000732792	17.31
	OsGRb09323	4	21564212	0.000733361	17.29
	OsGRg06481	4	21313607	0.000733361	17.20
	OsGRb08806	4	12650451	0.000733361	17.17
	OsGRb08813	4	12874705	0.000733361	17.16
	OsGRg06515	4	21663324	0.000741247	17.13
	OsGRb15879	8	5219860	0.000742324	17.13
	OsGRb08847	4	13340370	0.000756465	17.12
	OsGRb25737	4	10077235	0.000763724	17.10
	OsGRb08680	4	6440881	0.000764093	17.03
	OsGRb16283	8	10764889	0.000771753	17.00
	OsGRb08606	4	5500642	0.000784231	16.98

	OsGRb08627	4	5670305	0.000784231	16.96
	OsGRb08694	4	6660438	0.000784231	16.93
	OsGRb25632	4	4940344	0.000784231	16.92
	OsGRb09194	4	20010914	0.000784231	16.88
	OsGRb09207	4	20199885	0.000784231	16.87
	OsGRb09214	4	20274610	0.000784231	16.73
	OsGRg06440	4	20086815	0.000784231	16.71
	OsGRb15664	8	3149787	0.000792674	16.70
	OsGRb08808	4	12675840	0.000828223	16.70
	OsGRb09328	4	21610688	0.000833843	16.70
	OsGRb25901	4	21362199	0.000833843	16.70
	OsGRg06507	4	21623763	0.000833843	16.70
	OsGRb25899	4	20863673	0.000835161	16.70
	OsGRg06415	4	19655752	0.000840551	16.62
	OsGRb25775	4	11747654	0.000846295	16.62
	OsGRg06551	4	22587609	0.000867816	16.60
	OsGRb25803	4	14321111	0.0008711	16.58
	OsGRb08799	4	12535482	0.000885343	16.55
	OsGRg06453	4	20414472	0.000892554	16.53
	OsGRg06299	4	9673467	0.000895015	16.53
	OsGRb08990	4	17475598	0.00091055	16.53
	OsGRb08759	4	11454103	0.00091055	16.53
	OsGRb08766	4	11794996	0.00091055	16.53
	OsGRg06245	4	6939489	0.000910949	16.50
	OsGRb15974	8	6205816	0.000922788	16.47
	OsGRg06447	4	20096863	0.000922968	16.47
	OsGRb30636	4	20645999	0.000930739	16.46
	OsGRg06525	4	22150395	0.000933322	16.46
	OsGRg03752	2	34032581	0.000936312	16.46
	OsGRb09282	4	21089615	0.000939103	16.46
	OsGRb25626	4	4148271	0.000940647	16.46
	OsGRg06529	4	22194439	0.000945565	16.44
	OsGRb08633	4	5735183	0.000957862	16.43
	OsGRb09289	4	21225501	0.000957862	16.41
	OsGRb09307	4	21340929	0.000957862	16.37
	OsGRb31669	8	27620157	0.000959899	16.37

	OsGRg06450	4	20099643	0.000965278	16.37
	OsGRb14503	7	15365358	0.000975387	16.36
	OsGRb28608	9	13162604	0.000978713	16.33
	OsGRb09256	4	20715720	0.000990246	16.33
	OsGRb09232	4	20440235	0.000994596	16.33
	OsGRb08676	4	6365651	0.000995588	16.33
**1000 grain weight (TGW)**	OsGRb23906	1	10116371	0.000902976	15.63
	OsGRb05492	2	34355959	0.000148332	14.60
	OsGRg01164	1	28331892	0.000216037	13.83
	OsGRb21690	11	21789361	0.000222423	13.77
**Tiller/ plant (TP)**	OsGRb13190	6	20245648	0.00021125	17.57
	OsGRg04446	3	6518355	0.000107335	14.09
	OsGRg03402	2	28582430	0.000239771	12.44
	OsGRb29047	11	858733	0.000269943	12.19
**Unfilled Grains (UG/P)**	OsGRg07442	5	258353	0.000292806	15.74
	OsGRb14503	7	15365358	0.000324535	15.51
	OsGRg11329	8	3389509	0.00039926	15.05
	OsGRb15879	8	5219860	0.000399863	15.05
	OsGRb15974	8	6205816	0.000412558	14.98
	OsGRb15664	8	3149787	0.000440233	14.83
	OsGRb18405	9	21000940	0.00047682	14.66
	OsGRb16283	8	10764889	0.000508358	14.52
	OsGRb08910	4	14035735	0.000531266	14.42
	OsGRg12794	9	22384257	0.000534432	14.41
	OsGRb15653	8	3005090	0.000542166	14.38
	OsGRb15702	8	3385132	0.000567692	14.28
	OsGRb30591	4	12914840	0.00057091	14.26
	OsGRb15730	8	3554391	0.000586197	14.21
	OsGRb25803	4	14321111	0.000629133	14.05
	OsGRb14687	7	17400358	0.000642796	14.00
	OsGRb14703	7	17504411	0.000642796	14.00
	OsGRg10445	7	17412788	0.000642796	14.00
	OsGRb17690	9	10920756	0.000772503	13.61
	OsGRg11693	8	21450206	0.00094483	13.17
**Yield kg/Ha (Y/H)**	OsGRb20658	11	7220561	0.000495796	18.71
	OsGRb30591	4	12914840	0.00065155	18.15

	OsGRb08205	3	35394566	0.000651771	18.15
	OsGRb09180	4	19850601	0.000932963	17.42
	OsGRb23685	12	27209750	0.000953745	17.37
Yield kg/Plot (Y/P)	OsGRb01011	1	13770374	0.000594655	16.03
	OsGRb09543	4	27450829	0.000644405	15.86
	OsGRb31605	8	18321045	0.000823727	15.34
	OsGRg10392	7	15377693	0.000823727	15.34
	OsGRb14555	7	15910856	0.000953954	15.03
	OsGRg12112	9	5025651	0.000953954	15.03
	OsGRg07137	4	31075040	0.000981469	14.97

Supplementary Table S7: Identification of Candidate Genes corresponding to the significantlt associated SNPs.

SNP_name	chrnum	Strand	snp_position	region	Gene_ID
OsGRb30080	2	-	18212087	intergenic	Os02g0508500|45221;Os02g0510100|19402;Os02g0510300|27182
OsGRb14914	7	+	20814664	intron	Os07g0531700
OsGRb15039	7	-	22304042	3UTR	Os07g0558200
OsGRb05211	2	+	29829169	intron	Os02g0719000
OsGRb24510	2	-	9826527	CDS	Os02g0271600
OsGRb24572	2	-	12405692	intergenic	Os02g0313450|23371;Os02g0313700|537;Os02g0313900|8692
OsGRb24900	2	-	30132327	intergenic	Os02g0724100|32147;Os02g0724850|2226;Os02g0725100|7584;Os02g0725500|25276;Os02g0725700|31973
OsGRb04151	2	-	17990840	3UTR	Os02g0505700
OsGRb04250	2	-	19120460	intergenic	Os02g0523300|23066;Os02g0523500|14999;Os02g0523650|8186
OsGRg02806	2	+	17273659	intergenic	Os02g0492500|46331;Os02g0492800|28927;Os02g0493087|486
OsGRb04064	2	-	16775829	intergenic	Os02g0484975|24267
OsGRb04108	2	+	17345198	intergenic	Os02g0494600|2684;Os02g0494700|13518
OsGRb04456	2	-	21810979	CDS	Os02g0570700

OsGRb04559	2	+	22869364	CDS	Os02g0591500
OsGRb04569	2	+	23120515	intergenic	Os02g0595200|21927;Os02g0595700|6685;Os02g0595800|72;Os02g0596200|21596
OsGRb30142	2	-	24285137	CDS	Os02g0614800
OsGRb04511	2	+	22354743	intergenic	Os02g0580300|17067; Os02g0580600|2737;Os02g0580700|452;Os02g0580966|15583;Os02g0581000|26456;Os02g0581100|35630;Os02g0581200|46591
OsGRb16114	8	-	8615206	CDS	Os08g0241800
OsGRb16145	8	+	8929911	intergenic	Os08g0246300|9589;Os08g0246400|25383;Os08g0246500|33882;Os08g0246700|36393;Os08g0246800|39103
OsGRb04104	2	-	17309380	intron	Os02g0494000
OsGRb04421	2	-	20975538	intron	Os02g0555200
OsGRb04893	2	+	26259806	intergenic	Os02g0650800|21516; Os02g0650900|16826;Os02g0651200|12684;Os02g0651500|2841;Os02g0651900|11875
OsGRb21146	11	+	15034296	intergenic	Os11g0448700|36114; Os11g0449600|40946;Os11g0450050|44351
OsGRg02884	2	-	19568531	intron	Os02g0531600
OsGRg03095	2	+	23712290	CDS	Os02g0605000
OsGRb25451	3	+	24409509	intergenic	Os03g0637800|6375;Os03g0638200|39626
OsGRb21461	11	-	19084575	intergenic	Os11g0525500|27707;Os11g0525800|5273;Os11g0525900|716;Os11g0526800|28084;Os11g0527100|43579;Os11g0527300|49512
OsGRg14009	11	+	18935157	intergenic	Os11g0523700|9903;Os11g0524300|46738
OsGRb05101	2	-	28940961	CDS	Os02g0702500
OsGRg02717	2	-	11885546	intron	Os02g0304900
OsGRb04072	2	+	16904305	intergenic	Os02g0487300|11224
OsGRb04943	2	+	26640578	intergenic	Os02g0658150|31579;Os02g0658300|18687;Os02g0658600|590;Os02g0658800|8259;Os02g0659100|28218;Os02g0659500|48848
OsGRg03565	2	+	30862290	intron	Os02g0738950
OsGRb215	11	-	19994753	5UTR|CDS	Os11g0544200;Os11g0544200

48					
OsGRg02708	2	-	11594325	CDS	Os02g0301100
OsGRb25462	3	+	25181277	intergenic	Os03g0648600|45196;Os03g0648900|34885;Os03g0649050|30134;Os03g0650000|15780;Os03g0650350|46300
OsGRb25459	3	-	25131417	intron	Os03g0648500
OsGRg05363	3	+	24961621	intergenic	Os03g0645900|361;Os03g0646100|35026;Os03g0646300|42043
OsGRb21468	11	-	19129697	CDS	Os11g0527100
OsGRb21471	11	+	19170789	intergenic	Os11g0527000|42651; Os11g0527150|40433;Os11g0527500|22150;Os11g0527700|13663;Os11g0528200|2;Os11g0528300|2107;Os11g0528812|13584;Os11g0529150|36925
OsGRb32442	11	+	19026652	intergenic	Os11g0524300|38738; Os11g0525200|21914;Os11g0525600|31768;Os11g0525700|42569
OsGRg03058	2	+	23529294	intron	Os02g0601300
OsGRg03325	2	+	26931234	intron	Os02g0664000
OsGRb30127	2	+	22416322	CDS	Os02g0581400
OsGRb04643	2	+	23850363	5UTR|5UTR	Os02g0608100; Os02g0608100
OsGRg02730	2	+	12762219	intergenic	Os02g0319100|33346; Os02g0320000|6415;Os02g0320300|3063;Os02g0320600|15600;Os02g0320800|26449
OsGRb24567	2	-	12345631	intron	Os02g0312800
OsGRg13980	11	-	18173691	intron	Os11g0508600
OsGRb25578	3	+	33753141	intron	Os03g0807500
OsGRb09564	4	-	27939281	CDS	Os04g0557500
OsGRg04275	3	-	4267961	intron	Os03g0181550
OsGRb17703	9	-	11125119	CDS	Os09g0350900
OsGRb28603	9	+	12952275	intergenic	Os09g0381600|37451
OsGRb31905	9	-	17074373	intergenic	Os09g0453900|38940; Os09g0454200|6846;Os09g0454500|3292;Os09g0454900|17831;Os09g0455200|34916

OsGRb17510	9	+	7800022	3UTR	Os09g0305300
OsGRb28600	9	+	12915373	intergenic	Os09g0381100|26849; Os09g0381300|17367;Os09g0381600|549
OsGRg12308	9	+	14048196	CDS	Os09g0401100
OsGRg12310	9	+	14048809	CDS	Os09g0401100
OsGRb18178	9	-	19105152	intron	Os09g0493200
OsGRb27733	7	+	20438541	intergenic	Os07g0525400|19952; Os07g0525450|17375;Os07g0526150|17001
OsGRb14446	7	-	14594194	intergenic	Os07g0436100|13882; Os07g0436350|3483;Os07g0437000|24064
OsGRb30717	4	+	29956594	intergenic	Os04g0591232|34925; Os04g0591300|16643;Os04g0592400|8558;Os04g0593400|44940;Os04g0593500|47700
OsGRb09705	4	+	29950172	intergenic	Os04g0591232|28503; Os04g0591300|10221; Os04g0592400|14980
OsGRb31240	7	+	9363018	intergenic	Os07g0264000|13557; Os07g0264100|5314;Os07g0264800|32347;Os07g0264900|36333;Os07g0265100|42821
OsGRb12976	6	+	16465767	intergenic	Os06g0484400|35800; Os06g0484500|38747;Os06g0484600|43561
OsGRb13852	7	-	485365	intergenic	Os07g0108200|40529; Os07g0108300|34116;Os07g0108400|31655;Os07g0108500|25147;Os07g0108900|7710;Os07g0109100|11217;Os07g0109500|38401;Os07g0109600|43341
OsGRb13891	7	-	754356	CDS	Os07g0113700
OsGRg01923	1	+	39950738	intron	Os01g0916400
OsGRb13812	7	+	219361	3UTR	Os07g0103500
OsGRb14044	7	-	1929142	intron	Os07g0136500
OsGRb28184	8	-	14792948	intergenic	Os08g0333100|26682
OsGRb27102	6	-	24322609	intergenic	Os06g0609600|46656; Os06g0609700|40123;Os06g0609850|30126;Os06g0610100|22855;Os06g0610500|9406;Os06g0611100|41392;Os06g0611150|42642;Os06g0611200|47962
OsGRb27139	6	-	25535681	intergenic	Os06g0630850|10422
OsGRg05238	3	+	21673888	intron	Os03g0586900

OsGRb14503	7	-	15365358	intergenic	Os07g0447200|49709; Os07g0448300|3824;Os07g0448600|11682
OsGRb13785	7	+	20400	CDS|CDS	Os07g0100300; Os07g0100300
OsGRb23906	1	-	10116371	intron|intron	Os01g0283000; Os01g0283000
OsGRg09425	6	+	21482221	3UTR	Os06g0561000
OsGRb24377	1	-	40794243	3UTR	Os01g0929500
OsGRb10830	5	+	10350550	intergenic	Os05g0264200|6058
OsGRb30591	4	-	12914840	intergenic	Os04g0294401|3101; Os04g0294812|20440;Os04g0295100|40925
OsGRb08881	4	+	13725269	intergenic	Os04g0304400|49385; Os04g0304750|34299;Os04g0305700|46203
OsGRb08538	4	+	4440931	intron|intron	Os04g0165300; Os04g0165300
OsGRg06306	4	-	11320635	intron|intron	Os04g0271000; Os04g0271000
OsGRb25787	4	-	12725098	CDS	Os04g0291900
OsGRb09155	4	-	19209543	intergenic	Os04g0389901|28627; Os04g0391000|31202
OsGRb08910	4	-	14035735	intergenic	Os04g0309100|38178; Os04g0309400|29740;Os04g0309751|23242;Os04g0310100|3162;Os04g0310500|26921;Os04g0310800|36906
OsGRb25664	4	-	8124043	intron	Os04g0221600
OsGRb09180	4	+	19850601	intergenic	Os04g0401200|23; Os04g0401700|36073
OsGRg06407	4	-	19601384	intergenic	Os04g0396550|34615; Os04g0397100|82;Os04g0397500|11460;Os04g0397901|39416
OsGRg06430	4	+	19893385	intergenic	Os04g0401200|42807; Os04g0401700|1030;Os04g0402250|31023;Os04g0402300|39856
OsGRg06261	4	-	6968387	CDS	Os04g0202500
OsGRb25862	4	+	19570740	intron	Os04g0396500
OsGRb09200	4	-	20060586	intergenic	Os04g0403600|40187; Os04g0403701|35445;Os04g0403900|13669;Os04g0404100|2676;Os04g0404400|4612;Os04g0404900|23339;Os04g0405100|39579;Os04g0405300|48991

OsGRb09238	4	+	20485782	intergenic	Os04g0413900|41174; Os04g0414000|34166;Os04g0414100|22842;Os04g0414300|591;Os04g0414700|10272;Os04g0414800|13639;Os04g0415000|22843;Os04g0415100|33354;Os04g0415200|40328;Os04g0415600|48108
OsGRb09240	4	-	20505437	intergenic	Os04g0414250|34605; Os04g0414500|18351;Os04g0414850|4413;Os04g0415401|23348;Os04g0416100|44551
OsGRg06458	4	+	20426851	3UTR	Os04g0413500
OsGRb08754	4	-	11300762	intergenic	Os04g0269900|29232;Os04g0270200|6652;Os04g0270900|8718;Os04g0271000|15474
OsGRg06304	4	-	11316471	3UTR|3UTR	Os04g0271000; Os04g0271000
OsGRb25740	4	-	10304323	intergenic	Os04g0257500|12414
OsGRb08742	4	+	10624962	5UTR	Os04g0261400
OsGRb25839	4	+	17182231	intron	Os04g0359100
OsGRg06490	4	+	21414906	intron|intron	Os04g0432000; Os04g0432000
OsGRg06518	4	-	22025935	intron|intron	Os04g0442300; Os04g0442300
OsGRb17738	9	+	11674055	CDS	Os09g0359500
OsGRg11329	8	-	3389509	3UTR	Os08g0157900
OsGRb25742	4	-	10354477	intergenic	Os04g0257500|36863
OsGRb09195	4	+	20025177	intergenic	Os04g0403200|29019; Os04g0403300|15652;Os04g0403400|9648;Os04g0403500|5584;Os04g0404000|22399;Os04g0404601|40244
OsGRb09323	4	-	21564212	CDS	Os04g0433900
OsGRg06481	4	+	21313607	intron	Os04g0429800
OsGRb08806	4	+	12650451	CDS	Os04g0290800
OsGRb08813	4	-	12874705	intergenic	Os04g0294401|43236
OsGRg06515	4	-	21663324	CDS|CDS	Os04g0435500; Os04g0435500
OsGRb15879	8	-	5219860	intergenic	Os08g0190200|49057

OsGRb08847	4	+	13340370	intergenic	Os04g0300100|28399
OsGRb25737	4	+	10077235	intergenic	Os04g0254000|12026; Os04g0254300|1671
OsGRb08680	4	-	6440881	intergenic	Os04g0194000|13817; Os04g0194433|29727;Os04g0194500|34476
OsGRb16283	8	-	10764889	intergenic	Os08g0277300|38100; Os08g0278600|31180;Os08g0278900|45506
OsGRb08606	4	+	5500642	intergenic	Os04g0180400|13939; Os04g0180900|21271
OsGRb08627	4	-	5670305	5UTR	Os04g0183201
OsGRb08694	4	-	6660438	intergenic	Os04g0197275|22387; Os04g0197500|4642
OsGRb25632	4	+	4940344	3UTR	Os04g0172560
OsGRb09194	4	+	20010914	CDS	Os04g0403400
OsGRb09207	4	+	20199885	intergenic	Os04g0406600|28639; Os04g0407500|3615;Os04g0407800|747;Os04g0407900|11687
OsGRb09214	4	+	20274610	intergenic	Os04g0409600|18889; Os04g0409900|15337;Os04g0410300|1553;Os04g0410400|5750;Os04g0410700|8104;Os04g0411200|30895;Os04g0411300|35545
OsGRg06440	4	-	20086815	intron	Os04g0404900
OsGRb15664	8	+	3149787	3UTR	Os08g0154700
OsGRb08808	4	-	12675840	intergenic	Os04g0290450|45186; Os04g0290750|26641;Os04g0292150|42210;Os04g0291900|43210
OsGRb09328	4	-	21610688	intron	Os04g0434600
OsGRb25901	4	-	21362199	3UTR	Os04g0430800
OsGRg06507	4	-	21623763	intron|intron	Os04g0434800;Os04g0434800
OsGRb25899	4	+	20863673	intron	Os04g0421900
OsGRg06415	4	+	19655752	3UTR	Os04g0398000
OsGRb25775	4	+	11747654	5UTR	Os04g0278100
OsGRg06551	4	+	22587609	CDS	Os04g0452500

OsGRb25803	4	+	14321111	intergenic	Os04g0313600|35592;Os04g0314201|7716
OsGRb08799	4	-	12535482	intron	Os04g0288500
OsGRg06453	4	+	20414472	intron	Os04g0413200
OsGRg06299	4	-	9673467	intron|intron	Os04g0249600;Os04g0249600
OsGRb08990	4	-	17475598	intergenic	Os04g0363700|16019
OsGRb08759	4	+	11454103	5UTR	Os04g0272700
OsGRb08766	4	+	11794996	intron|intron	Os04g0278200;Os04g0278200
OsGRg06245	4	+	6939489	Intergenic	Os04g0202200|15025;Os04g0202700|35812
OsGRb15974	8	+	6205816	Intergenic	Os08g0205300|48039;Os08g0205400|44433;Os08g0205650|42758;Os08g0205800|36528;Os08g0206400|223;Os08g0206600|12872;Os08g0206700|20540;Os08g0206800|27691;Os08g0206900|31633;Os08g0207300|45537
OsGRg06447	4	+	20096863	Intergenic	Os04g0404000|48772;Os04g0404601|30394;Os04g0404800|13939;Os04g0405000|2732;Os04g0405150|3751;Os04g0405700|34420;Os04g0405800|42312
OsGRb30636	4	+	20645999	Intergenic	Os04g0417600|19928;Os04g0418000|7717;Os04g0418500|44868
OsGRg06525	4	+	22150395	Intron	Os04g0444200
OsGRg03752	2	-	34032581	intron|intron	Os02g0799100;Os02g0799100
OsGRb09282	4	+	21089615	Intergenic	NA
OsGRb25626	4	+	4148271	Intergenic	Os04g0160801|21579
OsGRg06529	4	-	22194439	5UTR	Os04g0445000
OsGRb08633	4	-	5735183	Intergenic	Os04g0183401|45983;Os04g0184250|3414;Os04g0184450|4253;Os04g0184900|43672
OsGRb09289	4	-	21225501	Intergenic	Os04g0428900|1907;Os04g0428950|7121;Os04g0429050|23364
OsGRb09307	4	+	21340929	CDS	Os04g0430400
OsGRb31669	8	+	27620157	CDS	Os08g0550600

OsGRg06450	4	+	20099643	CDS	Os04g0405000
OsGRb14503	7	-	15365358	Intergenic	Os07g0447200|49709;Os07g0448300|3824;Os07g0448600|11682
OsGRb28608	9	+	13162604	Intergenic	Os09g0384601|43740;Os09g0386450|14656;Os09g0386500|18726
OsGRb09256	4	-	20715720	Intergenic	Os04g0418300|24665;Os04g0419400|12414;Os04g0419750|26461;Os04g0419800|42038
OsGRb09232	4	+	20440235	Intergenic	Os04g0412700|47152;Os04g0412800|44955;Os04g0412900|34489;Os04g0413200|24995;Os04g0413500|13314;Os04g0413900|3055;Os04g0414000|6632;Os04g0414100|17727;Os04g0414300|43913
OsGRb08676	4	-	6365651	Intergenic	Os04g0193300|18986
OsGRb23906	1	-	10116371	intron|intron	Os01g0283000;Os01g0283000
OsGRb05492	2	-	34355959	CDS	Os02g0805250
OsGRg01164	1	+	28331892	intron|intron	Os01g0686800;Os01g0686800
OsGRb21690	11	-	21789361	Intergenic	Os11g0577300|26425;Os11g0577350|26083;Os11g0577675|8740;Os11g0577866|572;Os11g0578066|15406
OsGRb13190	6	-	20245648	Intergenic	Os06g0538900|20956;Os06g0539100|13974;Os06g0539500|11602;Os06g0540050|35029;Os06g0540200|36824
OsGRg04446	3	+	6518355	3UTR|3UTR|3UTR	Os03g0224200;Os03g0224200;Os03g0224200
OsGRg03402	2	-	28582430	CDS	Os02g0695800
OsGRb29047	11	+	858733	3UTR	Os11g0119200
OsGRg07442	5	+	258353	3UTR	Os05g0104700
OsGRb14503	7	-	15365358	intergenic	Os07g0447200|49709;Os07g0448300|3824;Os07g0448600|11682
OsGRg11329	8	-	3389509	3UTR	Os08g0157900
OsGRb15879	8	-	5219860	intergenic	Os08g0190200|49057
OsGRb15974	8	+	6205816	intergenic	Os08g0205300|48039;Os08g0205400|44433;Os08g0205650|42758;Os08g0205800|36528;Os08g0206400|223;Os08g0206600|12872;Os08g0206700|20540;Os08g0206800|27691;Os08g0206900|31633;Os08g0207300|45537
OsGRb15664	8	+	3149787	3UTR	Os08g0154700

OsGRb18405	9	-	21000940	intergenic	Os09g0533600|33488;Os09g0533800|25750;Os09g0533900|20289;Os09g0534000|14561;Os09g0535000|32163
OsGRb16283	8	-	10764889	intergenic	Os08g0277300|38100;Os08g0278600|31180;Os08g0278900|45506
OsGRb08910	4	+	14035735	intergenic	Os04g0309500|29465;Os04g0309600|22292;Os04g0309900|6548;Os04g0310200|4772;Os04g0310400|20800;Os04g0310900|37133
OsGRg12794	9	+	22384257	intron|3UTR	Os09g0563300;Os09g0563300
OsGRb15653	8	-	3005090	intergenic	Os08g0152300|3257;Os08g0152366|2181;Os08g0152500|4603;Os08g0152600|8717;Os08g0152800|17578;Os08g0152900|26192
OsGRb15702	8	-	3385132	CDS	Os08g0157800
OsGRb30591	4	-	12914840	intergenic	Os04g0294401|3101;Os04g0294812|20440;Os04g0295100|40925
OsGRb15730	8	+	3554391	intergenic	Os08g0160300|19490;Os08g0160600|7463;Os08g0160901|3791;Os08g0161100|21435
OsGRb25803	4	+	14321111	intergenic	Os04g0313600|35592;Os04g0314201|7716
OsGRb14687	7	+	17400358	intron	Os07g0479100
OsGRb14703	7	+	17504411	CDS|CDS	Os07g0481000;Os07g0481000
OsGRg10445	7	-	17412788	intron	Os07g0479300
OsGRb17690	9	-	10920756	intergenic	Os09g0346900|42803;Os09g0347500|12773;Os09g0347700|1365;Os09g0347900|7640;Os09g0348766|44108
OsGRg11693	8	+	21450206	intron	Os08g0440800
OsGRb20658	11	+	7220561	intergenic	Os11g0235250|23265;Os11g0235700|1114
OsGRb30591	4	-	12914840	intergenic	Os04g0294401|3101;Os04g0294812|20440;Os04g0295100|40925
OsGRb08205	3	-	35394566	CDS	Os03g0841900
OsGRb09180	4	+	19850601	intergenic	Os04g0401200|23;Os04g0401700|36073
OsGRb23685	12	+	27209750	3UTR	Os12g0634700
OsGRb01011	1	-	13770374	intergenic	Os01g0346700|8390;Os01g0347100|29581;Os01g0347200|37151
OsGRb09543	4	+	27450829	intergenic	Os04g0547600|40445;Os04g0547900|24660;Os04g0548000|21006;Os04g0548300|6323;Os04g0549350|43896;Os04g0549400|47281

OsGRb31605	8	-	18321045	CDS|CDS	Os08g0387700;Os08g0387700
OsGRg10392	7	+	15377693	intron	Os07g0448400
OsGRb14555	7	-	15910856	3UTR	Os07g0457200
OsGRg12112	9	-	5025651	intergenic	Os09g0265800|45677;Os09g0266100|28257;Os09g0266600|1433
OsGRg07137	4	-	31075040	CDS	Os04g0612800
